# Hierarchical Modeling of the Nonlinear Optical Response of Composite Materials Based on Tetrathiafulvalene Derivatives

**DOI:** 10.3390/molecules29163720

**Published:** 2024-08-06

**Authors:** Lucia Mydlova, Bouchta Sahraoui, Abdelkrim El-Ghayoury, Janusz Berdowski, Anna Migalska-Zalas, Malgorzata Makowska-Janusik

**Affiliations:** 1 Faculty of Science and Technology, Jan Dlugosz University, Al. Armii Krajowej 13/15, 42-200 Czestochowa, Poland; lucia.mydlowa@gmail.com (L.M.); j.berdowski@ujd.edu.pl (J.B.); a.migalska-zalas@ujd.edu.pl (A.M.-Z.); 2 LPHIA, University of Angers, Bd Lavoisier 2, 49045 Angers, France; bouchta.sahraoui@univ-angers.fr; 3 CNRS, University of Angers, MOLTECH-Anjou, Bd Lavoisier 2, 49045 Angers, France; abdelkrim.elghayoury@univ-angers.fr

**Keywords:** NLO, molecular dynamics, DFT calculations, TTF derivatives

## Abstract

The presented work concerns computational investigations of the physical properties of composite materials based on polymer matrix and nonlinear optical (NLO) active chromophores. The structural, electronic, and optical properties of selected tetrathiafulvalene (TTF)-based chromophores have been calculated using quantum chemical methods. The polymer matrix changes the physical properties of the inserted chromophores influencing their optical parameters. To explain the mechanism of the NLO signal occurrence from the composites based on poly(methyl methacrylate) (PMMA) matrix and TTF chromophores, their structures are modeled using the classical molecular dynamics. In consequence, the structural properties of the composites are discussed according to the NLO requirements. By developing the theoretical model based on a discrete multipole local field approach, the impact of polymer matrix on the optical properties of chromophores is explained.

## 1. Introduction

**Figure 1:**
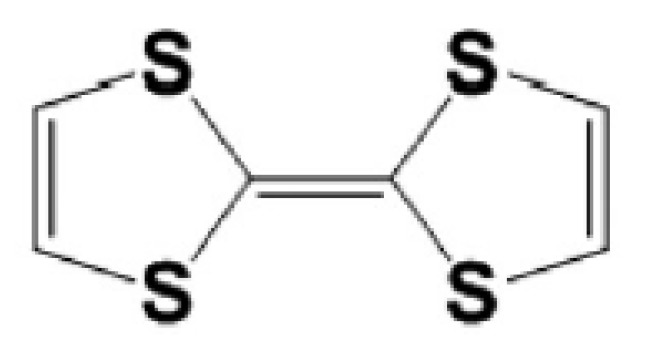
Chemical structure of the TTF moiety.

Organic push–pull molecules containing electron donor (D) and electron acceptor (A) moieties connected by the π-conjugated bridging group (D-π-A system) have properties required in telecommunication, optical computing, and optical signal processing [1,2]. Their unique properties are related to extended π-conjugated systems accompanied by moieties possessing strong donating characteristics, e.g., the sulfur heterocycle 2,2′-bis(1,3-dithiolylidene) (TTF) moiety [3,4] discovered by Wudl et al. in the early 1970s [5]. The TTF is composed of two five-membered heterocyclic rings and is characterized by strong π–π intermolecular interactions such as S•••S and C-H•••S (see Figure 1). It is well established that the TTF group and its numerous derivatives are excellent π-electron donors widely used in the preparations of molecular materials with various applications [6,7,8]. The TTF can easily be oxidized due to its low oxidation potential, resulting in mono-cationic (TTF^+^) and di-cationic (TTF^2+^) oxidized derivatives obtainable via two sequential and reversible oxidation processes [9]. Their oxidation potentials may be changed by the insertion of electron donors or electron acceptors according to the chemical tenability rule causing gentle molecular modifications [10]. The TTF is an appropriate heterocyclic system to build organic field-effect transistors (OFETs) [11]. In addition, TTF derivatives are also used as supramolecular photo-switchers [12], sensors [13], and storage devices [14]. Wartelle et al. [15] performed experimental and theoretical studies of the TTF properties examining, among others, charge transfer occurring in the TTF derivatives.

**Figure 2:**
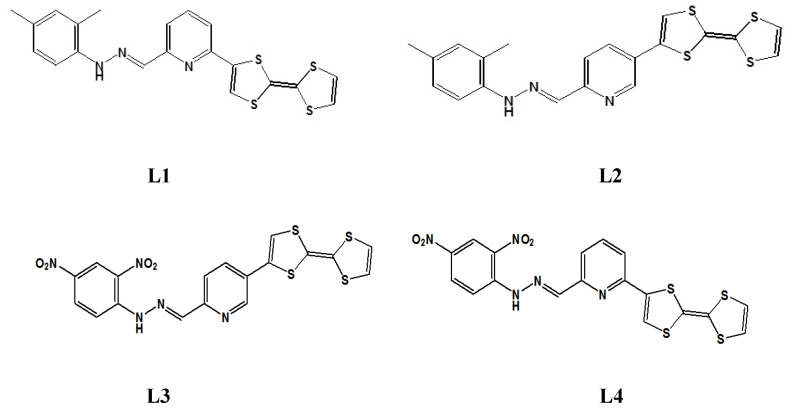
The structures of four investigated TTF-attended azine derivatives (LX) named L1, L2, L3, and L4.

In this work, we present a study of linear and nonlinear optical (NLO) properties of four newly synthesized TTF-appended azine derivatives, namely 2-([2,2′-bi(1,3-dithiolylidene)]-4-yl)-6-((2,4-dimethylphenyl)hydrazono) methyl)pyridine (L1), 5-([2,2′-bi(1,3-dithiolylidene)]-4-yl)-2-((2,4-dimethylphenyl)hydrazono) methyl)pyridine (L2), 5-([2,2′-bi(1,3-dithiolylidene)]-4-yl)-2-((2,4-dinitrophenyl)hydrazono) methyl)pyridine (L3), and 2-([2,2′-bi(1,3-dithioltlidene)]-4-yl)-6-((2,4-dinitrophenyl)hyrdazono) methyl)pyridine (L4), as presented in Figure 2, for developing a hierarchical methodology of computer simulations [16,17]. The investigated molecules are composed of the TTF moiety, acting as the donor part, and dimethylphenylhydrazone (also acting as a donor group) or dinitrophenylhydrazone, acting as an acceptor separated by the azine group, which can also be seen as an acceptor moiety. The great importance is the choice of the π-conjugated spacer playing a crucial role in the efficient electron communication between the donor and the acceptor parts. In our case, it is the pyridine moiety.

The proposed molecules L1, L2, L3, and L4, named LX, will be used as chromophores embedded in a poly(methyl methacrylate) (PMMA) polymer matrix. The PMMA can exist in both solid and liquid resin with excellent mechanical properties as well as optical and thermal stability. It is often used to construct thin film composites active in NLO devices. To explain the occurrence of the NLO properties of LX/PMMA composite systems, their structures were modeled using molecular dynamics (MD) with the density obtained from the experiment [18]. The electron and optical properties of LX chromophores were calculated by density functional theory (DFT). Then, the structural parameters of the simulated composites were used to investigate the influence of the PMMA polymer on the optical properties of chromophores. Because the experimental measurement of chromophores’ optical properties usually occurs in condensed phases, it is important to study how the surrounding environment influences the molecular NLO responses [19]. In this case, a hierarchical model based on a discrete approach was implemented to predict the optical properties of composite materials in bulk and thin film form.

There are essentially three major theoretical lines of development to include environmental effects in the quantum chemical models: continuum, where the solvent is considered implicitly as a polarizable medium characterized by its dielectric constant; discrete; and explicit approaches. In the discrete model, the solvent molecules with their recognized atoms are included along with the solute molecule, and the calculations are made for such molecular structures composed of the solute and a certain number of discrete solvent molecules treated as point charges [20]. The explicit approach treats the solvent and solute molecules as a discrete and explicit medium. The quantum chemical calculations should be performed for all atoms of the investigated system but it can be performed only at the level of the semiempirical methods or other not time-consuming approaches [21].

The discrete model was developed in different forms. One of them is based on quantum mechanics/molecular mechanics methodology (QM/MM) [22,23], where the average solvent electrostatic configuration (ASEC) approach is used [24]. This model is generally used to predict the properties of liquids where the solute molecule is of interest. The environmental interactions affecting the properties of molecules creating molecular crystals are considered by applying the local field theory introduced by Hurst and Munn [25,26]. In this approach, the local field at a molecule in the crystal is calculated rigorously as a sum of the external fields and the fields arising from all the other molecules in the crystal. It thereby allows us to express the macroscopic linear and nonlinear susceptibilities in terms of the molecular hyperpolarizabilities and the permanent electric field in the crystal [17,27,28]. In this case, the symmetry operators can be used to calculate the optical properties explicitly saving the time of calculations. The mentioned approach will be impossible to use for the amorphous polymer structures as the host-with-guest chromophores. In this case, the local field will be calculated explicitly from all atoms of the system modeled by MD. The local field added to the Hamiltonian will change the physical properties of the chromophore with respect to the isolated molecule, discussing the effect of the environment.

In the presented work, the hierarchical modeling approach based on MD followed by quantum chemical calculations by applying the discrete multipole local field approach was used to compare the linear and nonlinear optical properties of the chromophores in the vacuum and polymer matrix. The proposed model of hierarchical computer simulations is necessary to investigate the optical properties of composites. The developed approach can give the possibility to explain the nature of NLO properties of multi-component materials.

## 2. Results and Discussion

### 2.1. Structures of Composite Systems

**Figure 3:**
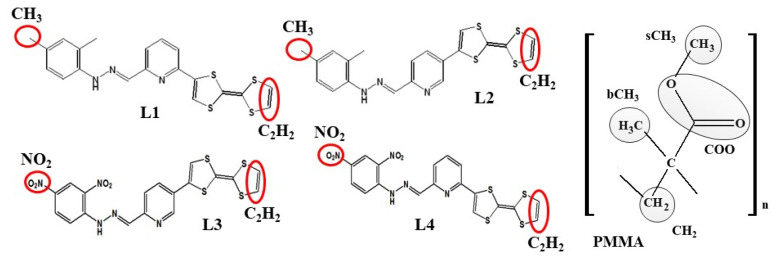
The structures of four investigated LX chromophores and mer of PMMA with marked groups of atoms used to construct the RDF function.

The spatial distribution of the chromophores embedded into the polymer matrix was examined using the intermolecular radial distribution function (RDF). The RDFs were calculated between the center of mass (COM) of selected groups of atoms located at chromophores and polymer, according to the following formula:

(1) $$G_{AB}\left(r\right)=\frac{\langle \rho_{B}(r)\rangle }{{\langle \rho_{B}\rangle }_{loc}}=\frac{1}{{\langle \rho_{B}\rangle }_{loc}}\frac{1}{N_{A}}\sum_{i\in A}^{N_{A}}\sum_{j\in B}^{N_{B}}\frac{\delta (r_{ij}-r)}{4\pi r^{2}}$$

where <*ρ_B_*(*r*)> is the density of group B at distance r around group A, and <*ρ_B_*>*_loc_* is the density of group B averaged over the sphere around group A with a radius of half the unit cell length. Two opposite-located groups of chromophore atoms were chosen for RDFs (see Figure 3). They are C_2_H_2_ atoms located on the (1,3-dithiol-2-one) group of TTF moiety and one of the methyl groups (CH_3_) from the 2,4-dimethyl phenyl moiety of the L1 and L2 chromophores, and one of the nitro groups (NO_2_) located on the 2,4-dinitro phenyl moiety of the L3 and L4 molecules. In the PMMA mer, four groups were chosen: the CH_2_, bCH_3_ (methyl group bonded to the backbone of an α-carbon atom), sCH_3_ (methyl group at the end of side group), and COO group. The COMs of the different groups were computed for each snapshot.

**Figure 4:**
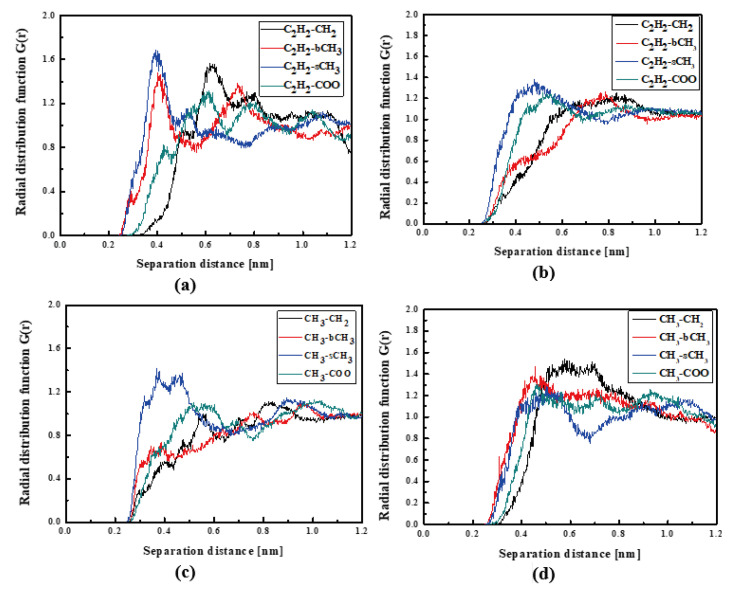
Partial RDFs calculated for distances between the center of mass of different moieties of the L1 molecule and different subunits of PMMA at T = 500 K (**a**,**c**) and T = 300 K (**b**,**d**) for the bulk L1/PMMA system. Panels (**a**,**b**) present the RDFs for C_2_H_2_ of L1 molecule and different subunits of PMMA mer. Panels (**c**,**d**) present the RDFs for CH_3_ of L1 and different subunits of PMMA mer.

In Figure 4, the RDFs calculated for the L1 chromophore and different groups of PMMA mer in the bulk form are presented. From Figure 4a, one can see that in a liquid state (at 500 K), the distance of C_2_H_2_-bCH_3_ is equal to 0.3 nm. The same distance is noticed for the CH_3_-bCH_3_ and CH_3_-sCH_3_ (see Figure 4c). It means that at 500 K, the chromophores L1 enter between side mer groups of the PMMA. In the glassy state (at 300 K), both groups of L1 are located close to the sCH_3_ side groups of the polymer (see Figure 4b,d). It means that the solidification of the L1/PMMA composite twists of the polymer and chromophores come out from between the mers, slightly moving away from them. At 300 K, the free volume around L1 chromophores has a diameter of 0.4 nm. The same distance is obtained for the host–guest polymer systems based on the PMMA matrix doped with different chromophores, namely N,N-dimethylp-nitroaniline (DPNA), 4-(dimethylamino)-4′-nitrostilbene (DMANS), and N,N′-di-*n*-propyl-2,4-dinitro-1,5-diaminobenzene (DPDNDAB) [29]. This is probably an intrinsic property of the polymer matrix.

**Figure 5:**
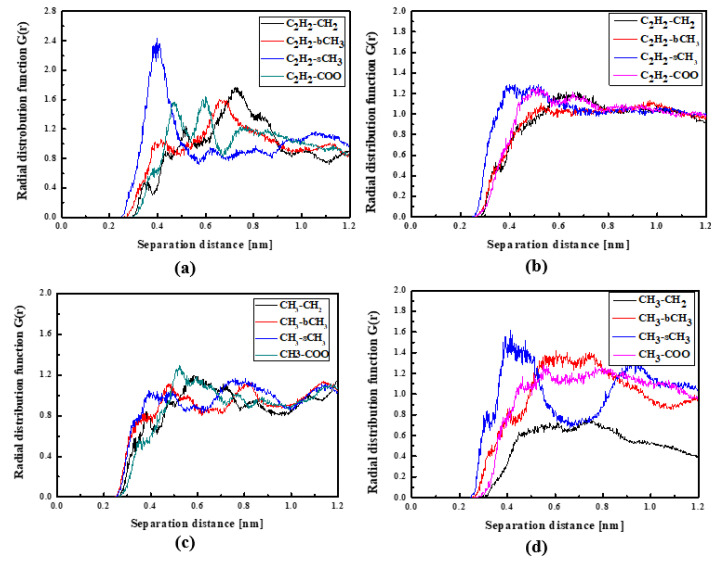
Partial RDFs calculated for distances between the center of mass of different moieties of the L2 molecule and different subunits of PMMA at T = 500 K (**a**,**c**) and T = 300 K (**b**,**d**) for the bulk L2/PMMA system. Panels (**a**,**b**) present the RDFs for C_2_H_2_ of L2 molecule and different subunits of PMMA mer. Panels (**c**,**d**) present the RDFs CH_3_ of L2 and different subunits of PMMA mer.

The RDFs calculated for the L2/PMMA composites do not have a shape describing a well-defined position of the L2 molecules in a polymer matrix, except for one arrangement shown in Figure 5a, presenting the distance for C_2_H_2_-sCH_3_. In the liquid state, the L2 molecule is located at 0.4 nm from the PMMA chain. It confirms that the polymer chain is curled up with side groups facing outward. When the L2/PMMA composite is solidified, the CH_3_ groups of L2 are the closest located to the sCH_3_ groups of the PMMA chain (see Figure 5d) and the distance is also close to 0.4 nm. One cannot see any evident difference between the location of the L1 and L2 molecules in the PMMA matrix.

**Figure 6:**
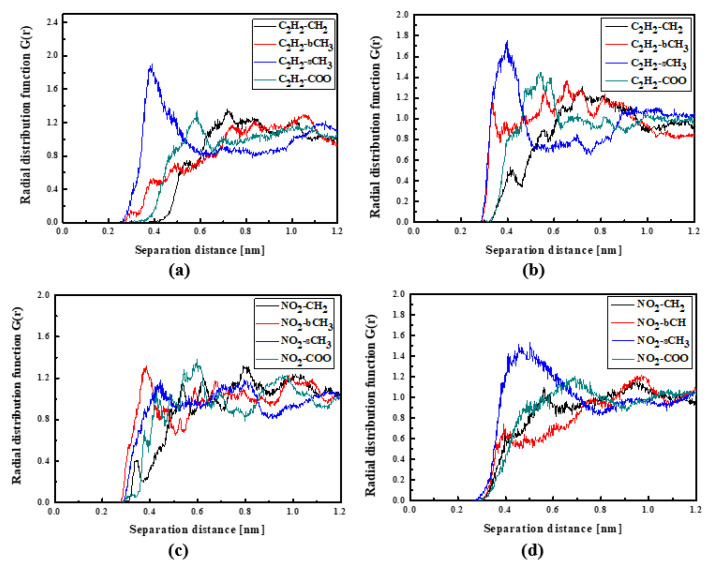
Partial RDFs calculated for distances between the center of mass of different moieties of the L3 molecule and different subunits of PMMA at T = 500 K (**a**,**c**) and T = 300 K (**b**,**d**) for the bulk L3/PMMA system. Panels (**a**,**b**) present the RDFs for C_2_H_2_ of the L3 molecule and different subunits of PMMA mer. Panels (**c**,**d**) present the RDFs NO_2_ of L3 and different subunits of PMMA mer.

The L3 chromophores are located closer to the PMMA chain than the L1 and L2 chromophores. In liquid (see Figure 6a) and in the glassy state (see Figure 6b), the L3 molecules are inverted to possess the TTF group close to the bCH_3_ group of PMMA. This behavior is significantly visible for the system in the glassy state. There are also some examples that the NO_2_ groups are located close to the backbone of the polymer chain (see Figure 6c). It means that the L3 chromophores enter into the polymer structure in the liquid state, locating themselves between the mers. This is also seen for the L3 chromophores in the glassy polymer (Figure 6b). 

**Figure 7:**
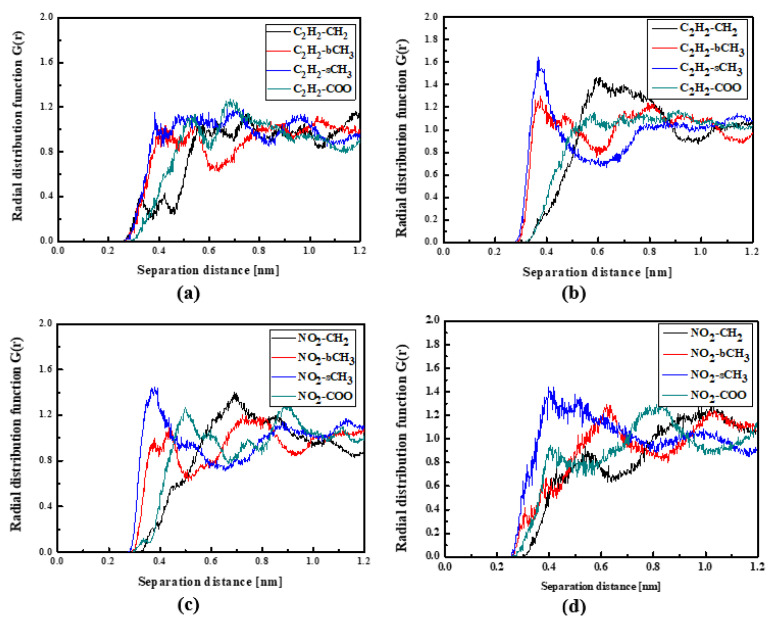
Partial RDFs calculated for distances between the center of mass of different moieties of the L4 molecule and different subunits of PMMA at T = 500 K (**a**,**c**) and T = 300 K (**b**,**d**) for the bulk L4/PMMA system. Panels (**a**,**b**) present the RDFs for C_2_H_2_ of the L4 molecule and different subunits of PMMA mer. Panels (**c**,**d**) present the RDFs NO_2_ of L4 and different subunits of PMMA mer.

In the case of the L4/PMMA composites, one can see chromophores located at 0.3 nm from the polymer chain. The TTF groups are close to the CH_2_ groups of the polymer (Figure 7a). As was observed for the L3/PMMA structure, here, the chromophores also enter into the structure of the polymer. In the glassy form of the L4/PMMA composites, their chromophores are at 0.3 nm from the polymer. One can conclude that the chromophores possessing the 2,4-dinitrophenyl group tend to be located closer to the PMMA chain than the chromophores with the 2,4-dimethylphenyl group. It was confirmed that the meta-position of the TTF group on the pyridine tended to be the closer to the location of the chromophores (L2 and L3) to the PMMA polymer chain than the other ones (L1 and L4).

**Figure 8:**
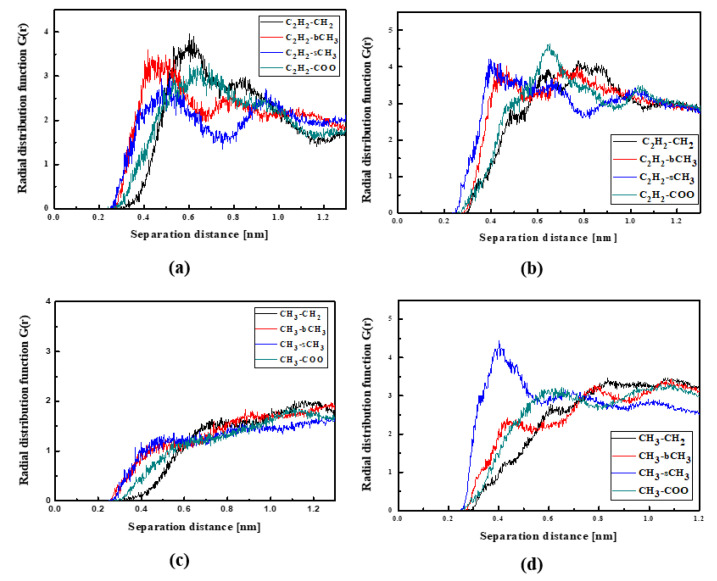
Partial RDFs calculated for distances between the center of mass of different moieties of the L1 molecule and different subunits of PMMA at T = 500 K (**a**,**c**) and T = 300 K (**b**,**d**) for the thin film L1/PMMA system. Panels (**a**,**b**) present the RDFs for C_2_H_2_ of L1 molecule and different subunits of PMMA mer. Panels (**c**,**d**) present the RDFs CH_3_ of L1 and different subunits of PMMA mer.

The NLO devices are generally constructed in the form of thin films where the surface behavior determines the material properties. Therefore, the influence of the surface on the structural and dynamic properties of the LX/PMMA composites was investigated. The RDFs created for the L1/PMMA system in the thin film form are presented in Figure 8. One can see that the distance between the C_2_H_2_ and bCH_3_ groups calculated for the film at 500 K is higher than observed for the bulk system (see Figure 4a). In liquid and solid states, the mentioned distance is equal to 0.4 nm (see Figure 8a,b). Distances between CH_3_ groups of the L1 and the selected groups of PMMA in liquid state are not well defined because the RDF peaks are broad. It means that the methyl groups of the L1 molecules are very mobile when the thin film system is liquid. When the system is solidified, both ends of chromophores are at 0.4 nm from the sCH_3_ group of PMMA (see Figure 8d). The presented RDFs allow us to conclude that the polymer surface is less dense than the volumetric composite and the chromophores L1 are farther away from the polymer. The free volumes created around chromophores located at the surface will allow for easier molecular reorientation necessary during the corona poling process used in NLO device construction. 

**Figure 9:**
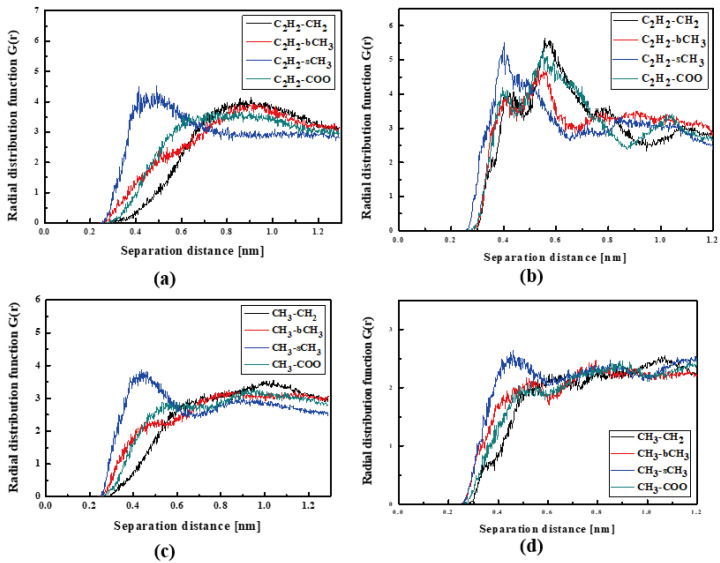
Partial RDFs calculated for distances between the center of mass of different moieties of the L2 molecule and different subunits of PMMA at T = 500 K (**a**,**c**) and T = 300 K (**b**,**d**) for the thin film L2/PMMA system. Panels (**a**,**b**) present the RDFs for C_2_H_2_ of L2 molecule and different subunits of PMMA mer. Panels (**c**,**d**) present the RDFs CH_3_ of L2 and different subunits of PMMA mer.

The L2 molecules embedded into PMMA in thin film form are farther from the polymer than previously noticed for the bulk L2/PMMA system. In this case, the TTF group of the L2 is the closest (0.4 nm) to the sCH_3_ groups of PMMA (see Figure 9a). When the thin film is solidified (Figure 9b), the TTF group of the L2 molecule enters between the mers. In this case, the distance between the C_2_H_2_ of the L2 and all selected groups of PMMA is the same and equal to 0.4 nm. The opposite side of the L2 molecule (the 2,4-dimethylphenyl group) is also located close to the sCH_3_ group (at 0.4 nm). 

**Figure 10:**
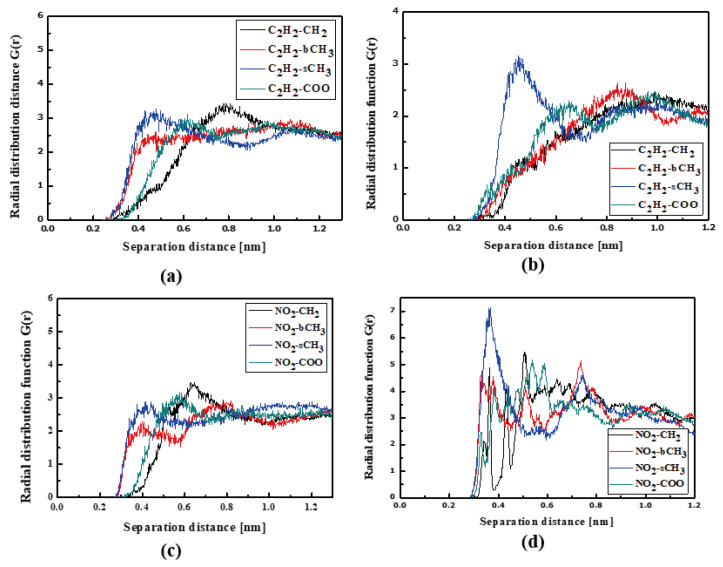
Partial RDFs calculated for distances between the center of mass of different moieties of the L3 molecule and different subunits of PMMA at T = 500 K (**a**,**c**) and T = 300 K (**b**,**d**) for the thin film L3/PMMA system. Panels (**a**,**b**) present the RDFs for C_2_H_2_ of the L3 molecule and different subunits of PMMA mer. Panels (**c**,**d**) present the RDFs for NO_2_ of L3 and different subunits of PMMA mer.

**Figure 11:**
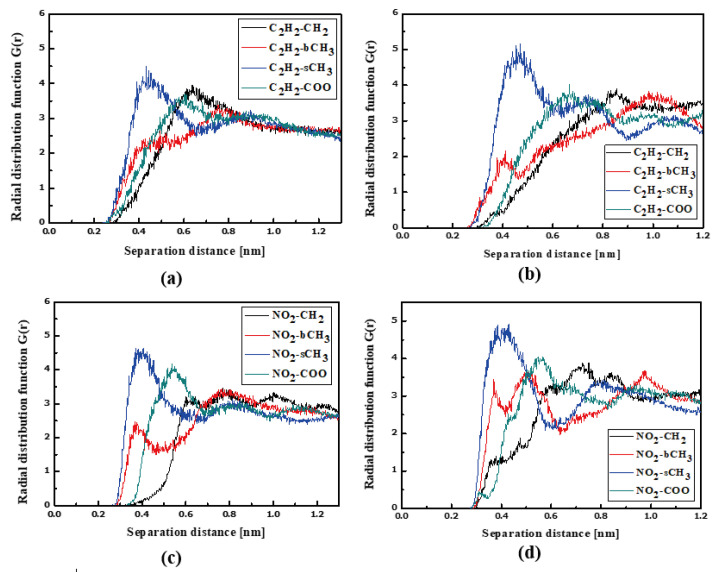
Partial RDFs calculated for distances between the center of mass of different moieties of the L4 molecule and different subunits of PMMA at T = 500 K (**a**,**c**) and T = 300 K (**b**,**d**) for the thin film L4/PMMA system. Panels (**a**,**b**) present the RDFs for C_2_H_2_ of the L4 molecule and different subunits of PMMA mer. Panels (**c**,**d**) present the RDFs of NO_2_ of L4 and different subunits of PMMA mer.

The RDFs of the L3/PMMA thin film are presented in Figure 10. The TTF groups are at a distance of 0.4 nm from the sCH_3_ and bCH_3_ of PMMA in a liquid state. The same is noticed for the NO_2_ group. The solidification does not change this position of the NO_2_ group but the TTF group is the closest to the sCH_3_ group (see Figure 10b,d). It means that the chromophores leave the inter-mer space during solidification. The distance between the L3 and polymer is equal to 0.4 nm. The L4 chromophores are located close to the sCH_3_ group of the polymer in thin film form (see Figure 11). It is true for the liquid and solid L4/PMMA forms. For the liquid and glassy state of the L4/PMMA, a small peak appears for the distance between the bCH_3_ and NO_2_ groups (see Figure 11c,d). It means that the chromophores enter into the polymer structure but the distance between the molecules is equal to 0.4 nm. 

Analyzing the data presented in this paragraph, one may conclude that around the chromophores embedded into the PMMA matrix, the free volume is created. It allows for the rotation of chromophores during the corona poling process. The intrinsic property of the PMMA allows us to create the free space around the LX molecules to be equal to 0.4 nm. It is in agreement with our previous work [29,30]. The performed MD simulations show that in bulk LX/PMMA guest–host systems, chromophores can appear closer to the sCH_3_ group, and the distance is insignificantly less than 0.4 nm. nm. Additionally, it was proven that the L3 and L4 molecules locate themselves close to the bCH_3_ groups of PMMA. It means that they enter between mers, causing difficulties during molecular reorientation. Additionally, one can conclude that the investigated composites are less dense at the surface than is observed in the bulk material.

### 2.2. Reorientation of Chromophores in PMMA Matrix

**Figure 12:**
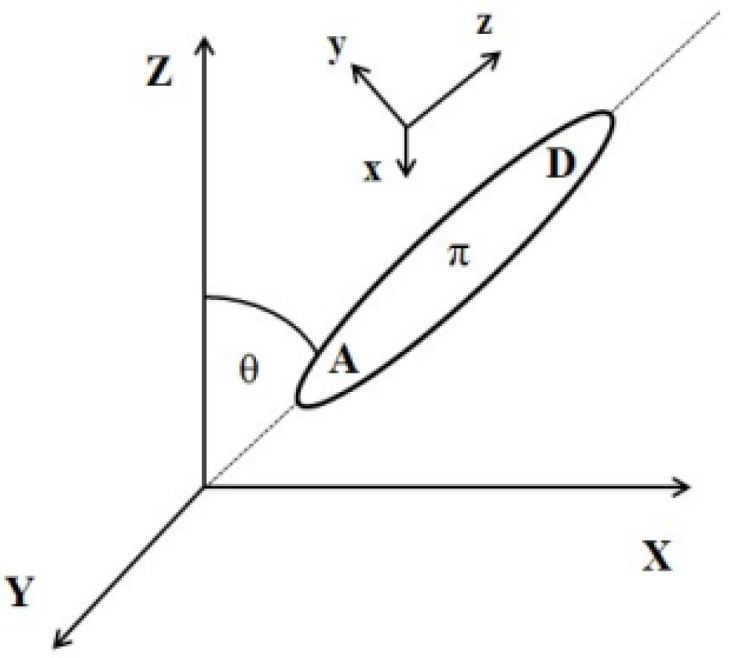
Relationship between laboratory and molecular coordinate systems.

The external electric field-induced reorientation of the chromophores was investigated by computing the <cosθ(t)> based on the angle between the dipole moment of the chromophore and the vector of the external electric field. The degree of alignment depends on the electric field strength and the spatial extension of the dipolar molecules. The idea of the cosθ(t) angle is presented in Figure 12. Values of <cosθ(t)> have been computed by averaging over three equivalent structures modeled for each of the LX/PMMA composites.

**Figure 13:**
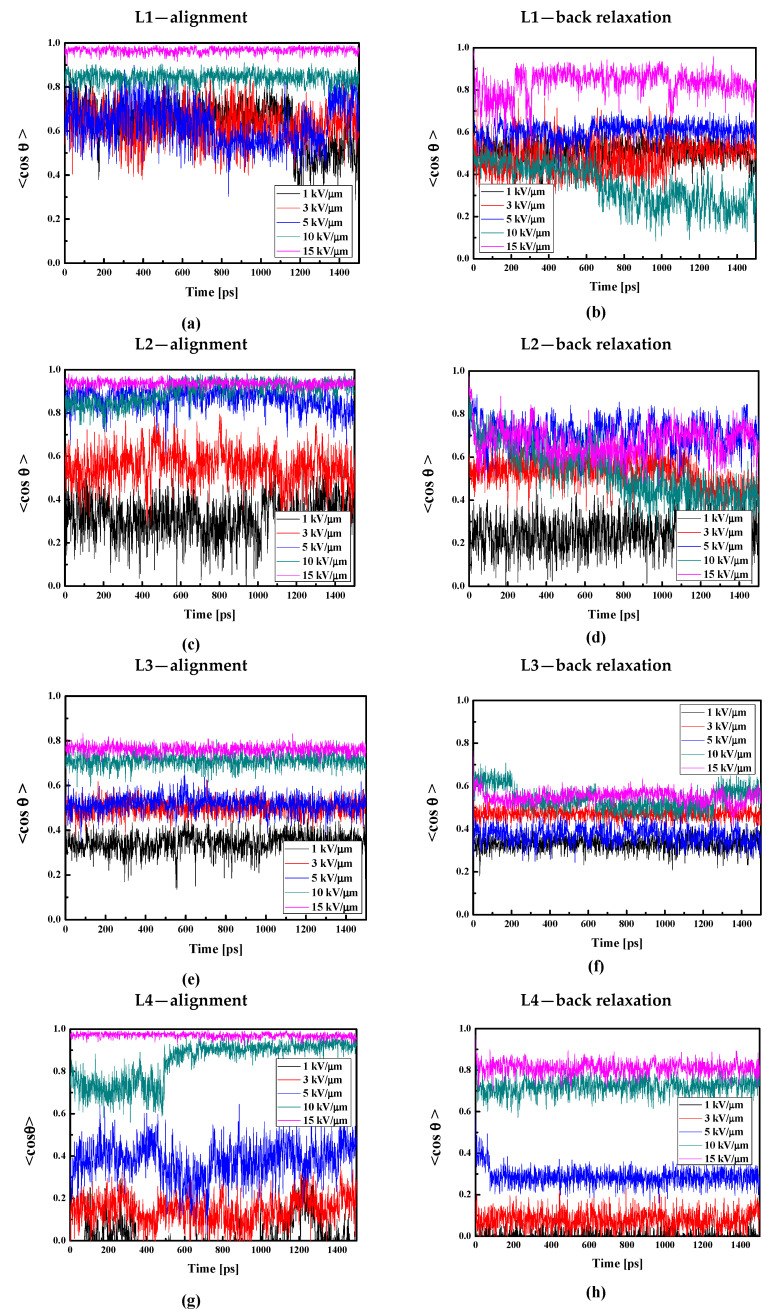
Changes in the value of the order parameter <cosθ(t)> versus the time of simulation and applied external electric field calculated by the MD technique for the L1/PMMA (**a**,**b**), L2/PMMA (**c**,**d**), L3/PMMA (**e**,**f**), and L4/PMMA (**g**,**h**) composites in the volumetric form at temperatures of 500 K (**a**,**c**,**e**,**g**) and 300 K after the simulated annealing without external electric field (**b**,**d**,**f**,**h**).

The values of the used external electric field were selected according to our previous work [29]. The L1/PMMA composite in bulk form exhibits the complete alignment by applying an external electric field equal to 15 kV/μm (see Figure 13a). Using the field 10 kV/μm, the system is aligned at 80% but using the electric field equal to 1, 3, or 5 kV/μm, the chromophores are aligned at 60% (see Figure 13a). The used intensities of the poling electric field are high compared to the experiment, but we should note that the MD process is very short compared to the real corona poling process; therefore, the field used in computer simulations should be correspondingly larger.

The full alignment of the L2 molecules in the PMMA bulk polymer can be obtained using an electric field with an intensity of 10 kV/μm (see Figure 13c). The field 5 kV/μm aligns L2 molecules in 80%. It means that the alignment of the chromophores L2 is easier than the alignment of the L1 molecules. However, the electric field with an intensity of 1 kV/μm is too small to move the L2 chromophores. The molecules L2 are closer to the polymer than the L1, and in consequence, the force to move L2 should be higher than the one applicable for the L1. With the stronger electric field, the movement of the polymer side groups close to L2 promotes the reorientation of chromophores, which is why it is easier to rotate the L2 than the L1 chromophores. Additionally, it is worth reminding that the L2 chromophores do not enter between the mers of PMMA.

The molecules L3 and L4 are significantly more difficult to align than the L1 and L2 chromophores. In the case of L3, an electric field with intensities of 10 and 15 kV/μm aligns the molecules at 80%. The alignment at the level of 50% is possible using an electric field equal to 3 and 5 kV/μm (see Figure 13e). The chromophore L4 embedded in the PMMA bulk polymer can be aligned by the electric field no less than 10 kV/μm (see Figure 13g). The chromophores located between the polymer side groups are more difficult to align because of the intermolecular interaction occurring between the chromophores and the polymer. This situation occurs despite the fact that the molecules L3 and L4 possess much higher electric dipole moments than the L1 and L2 chromophores (this will be discussed in the next paragraph), and their interaction with the external electric field is much higher. The L3 and L4 molecules enter deeper into the polymer structure, localizing in polymer cages and with more difficult rotation.

It should be noted that the poling external electric field was acting on the chromophores during cooling the system from 500 K up to 300 K. Then, the external electric field was switched off and the composites were modeled under an NVT ensemble to investigate their back relaxation. The <cosθ(t)> versus the time for the bulk LX/PMMA composites in the glassy state is presented in Figure 13. Analyzing these data, one can see that not only are the L4 chromophores relatively difficult to align, but their back relaxation is also prevented (see Figure 13h). The modeled L4 chromophores are still aligned at 80% after 1.5 ns of MD without an external electric field. At the same time, the alignment of the L3 molecules in PMMA decreases up to 50–60% (Figure 13f). One can see that the order of stability of the L1/PMMA and L2/PMMA is unsatisfactory. However, the L2 molecules keep the order parameter at the level of 70%. 

**Figure 14:**
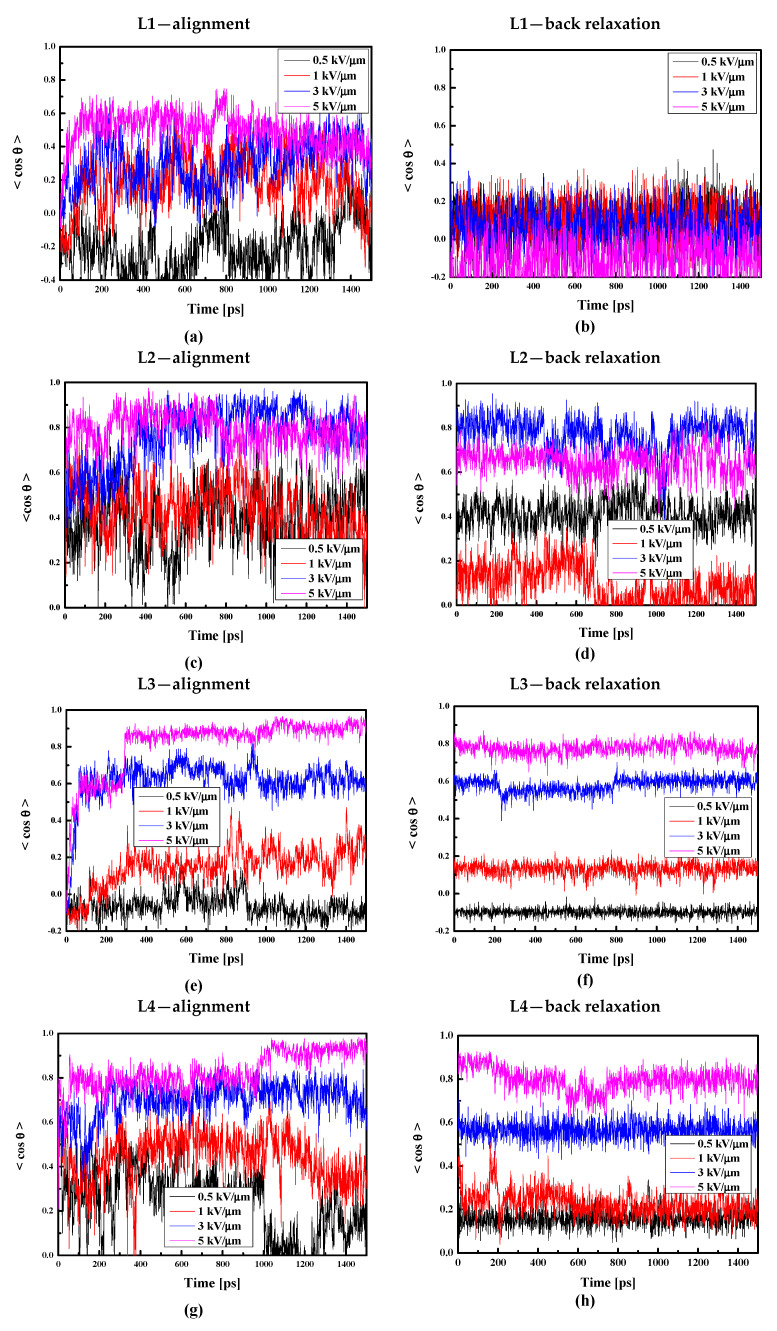
Changes in the value of the order parameter <cosθ(t)> versus the time of simulation and applied external electric field calculated by the MD technique for the L1/PMMA (**a**,**b**), L2/PMMA (**c**,**d**), L3/PMMA (**e**,**f**), and L4/PMMA (**g**,**h**) composites in the thin film form at the temperature of 500 K (**a**,**c**,**e**,**g**) and in glassy state (300 K) after the simulated annealing (**b**,**d**,**f**,**h**).

Additionally, taking into consideration that the thin film is less dense than the bulk composite, we decided to align the thin film composites by electric fields with intensities equal to 0.5, 1.0, 3.0, and 5.0 kV/μm. The obtained results are presented in Figure 14. Comparing data presented in Figure 13a and Figure 14a, one can conclude that it is easier to align chromophore L1 in liquid bulk PMMA polymer than in thin film form. This is not the case for the L2/PMMA. The surface limitation does not have any influence on the alignment of the L2 molecules in PMMA (see Figure 14c). Contrary to the L1 and L2, it is much easier to align the L3 chromophores in the thin film of the PMMA than in the bulk matrix. In this case, the electric field equal to 5 kV/μm and 3 kV/μm align the L3 chromophores at the level of 90% and 60%, respectively (see Figure 14e). The same situation is noticed for the L4/PMMA thin film system (see Figure 14g).

Analyzing the back relaxation of the LX chromophores in the thin film of PMMA, one can conclude that the L1/PMMA system is completely disordered after the back relaxation process. Relatively rigid is the L3/PMMA system. Also, the systems L2/PMMA and L4/PMMA are not distorted after relaxation without an external electric field. The obtained data allow us to conclude that the L1/PMMA composite is not suitable for first-order NLO applications.

### 2.3. Structural, Electron, and Optical Properties of TTF Derivatives 

Two conformers of each LX molecule identified as **a** and **b** were found (see Table 1). As was reported in our previous work, the existence of both conformers was proven experimentally [31]. Molecules depicted as **a** are more elongated than the molecules depicted as **b** [18]. The TTF group attached to the pyridine in the orto-position (L1^b^ and L4^b^) is more likely to twist the molecule in conformer **b** than the one attached in the meta-position (L2^b^ and L3^b^). In Table 1, the frontier molecular orbitals of LX molecules in both conformers calculated by the DFT/B3LYP method are collected. Analyzing the highly occupied molecular orbital (HOMO) and lowest unoccupied molecular orbital (LUMO) spreading out, one can conclude that all LX molecules possess donor-π-acceptor character with TTF moiety as a donor. The LUMO orbital, in the case of the weak acceptor 2,4-dimethylphenyl group, is dispersed on the whole molecule with a strong location on the azine-accepting group. This can be seen in the LUMOs of L1 and L2 molecules. In the case of the L3 and L4 molecules containing the 2,4-dinitrophenyl accepting group, the LUMO is mainly located on the dinitrophenyl accepting group with a contribution from the azine group.

The electron properties of the LX molecules were also calculated by the DFT/LC-BLYP/6-311++G** method. The energy difference between frontier orbitals (ΔE_HOMO-LUMO_) calculated for the LX molecules using both functionals are collected in Table 2. The data obtained by the DFT/LC-BLYP/6-311++G** method are similar to the ones calculated by the ab initio method and presented in our previous work [18]. The ΔE_HOMO-LUMO_ values calculated by using both functionals, B3LYP and LC-BLYP, have the same tendency. However, ΔE_HOMO-LUMO_ values obtained by using the DFT/B3LYP method are much lower compared with the ones calculated by the DFT/LC-BLYP method. This situation is in line with the assumptions of the DFT methodology, giving higher values of the ΔE_HOMO-LUMO_ using long correlated functionals. Additionally, one can mention that the 2,4-dinitrophenyl moiety decreases the value of the ΔE_HOMO-LUMO_, increasing the electric dipole moment of the L3 and L4 molecules in both conformers. The functional used does not influence the observed relationship. The obtained data favor the L3 and L4 molecules for the NLO applications.

Positions of the first absorption peaks calculated for the LX isolated molecules (in vacuum) and molecules in dichloromethane using the DFT/B3LYP and DFT/LC-BLYP methods are presented in Table 2. One can see that the DFT/B3LYP method shifts the λ_max_ values into the long-wavelength region compared with the DFT/LC-BLYP obtained results for all molecules. It is in agreement with the methodological reason. Dichloromethane shifts the λ_max_ for all molecules into the blue side of the spectrum (hypsochromic shift). It means that the first absorption peak is created by the n→π* transition. It is especially seen for the molecules containing the 2,4-dimethylphenyl group (L1 and L2) in both conformers (see Table 2). One can also conclude that the investigated molecules are more polar in the ground state than in the excited state [32]. In this case, the non-bonding electrons in the ground state are relatively stabilized by electrostatic interaction with the polar solvent. As a result, the first absorption peak is shifted to a shorter wavelength with increasing solvent polarity. The mentioned phenomenon was also checked for tetrahydrofuran (THF) and 1,1,2-trichloroethane (TCA) solvents but the obtained results are not presented here.

**Figure 15:**
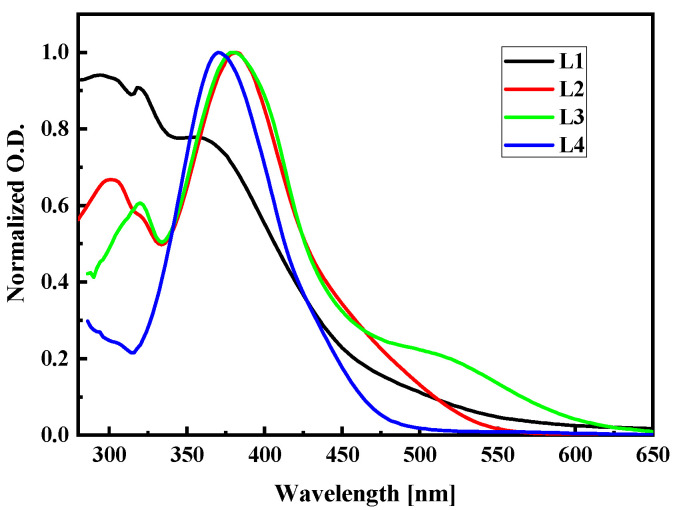
Experimental UV-vis absorption spectra of L1, L2, L3, and L4 chromophores measured in dichloromethane (~C = 2.6 × 10^−5^ M) at room temperature.

To check the correctness of the used computational methods and choose the one to calculate the NLO properties of the LX chromophores in the PMMA matrix, the UV-vis absorption spectra were studied. The UV-vis spectra of LX molecules dissolved in dichloromethane (~C = 2.6 × 10^−5^ M) were measured at room temperature in quartz cuvettes using the Perkin Elmer spectrophotometer and they are presented in Figure 15. The L1, L2, L3, and L4 molecules were synthesized according to procedures described in the work [18,33]. The highest wavelength value of the first absorption peak is noticed for the L3 molecule. Comparing the spectra of L2 and L3 molecules, one may conclude that the 2,4-dinitrophenyl group shifts the first absorption band into a higher wavelength, creating an additional peak in the case of the L3 at the red spectrum side. It is also seen by comparing the spectra of the L1 and L4 molecules. The strong electron absorption band exhibiting for the L1, L2, and L4 molecules centered at 350–400 nm is assigned to the n→π* transitions. The L3 molecules absorb light at the 500 nm assigned to the n→π* transition and at the 375 nm where the π→π* transition is observed.

**Figure 16:**
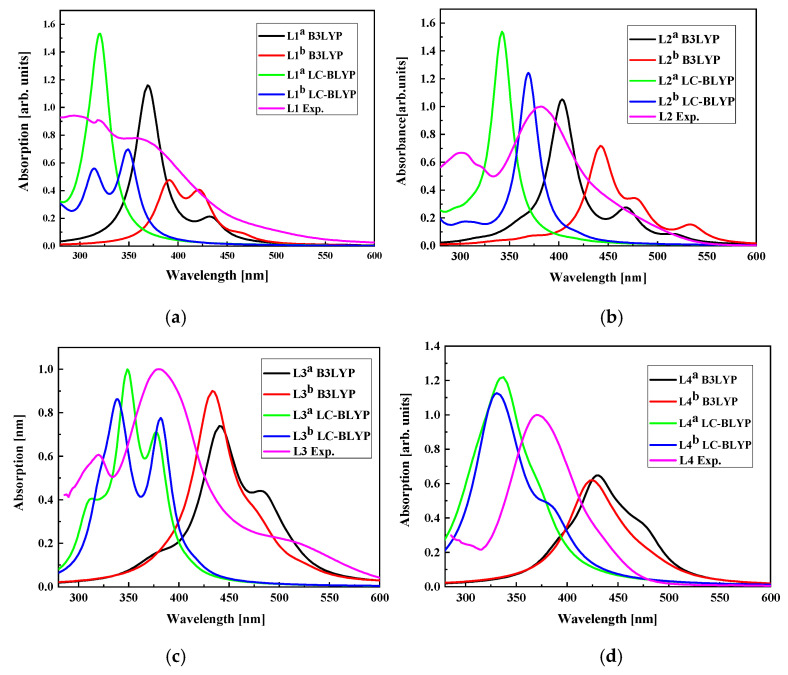
The UV-vis absorption spectra calculated by DFT/B3LYP and DFT/LC-BLYP methods for L1 (**a**), L2 (**b**), L3 (**c**), and L4 (**d**) molecules in dichloromethane compared with experimentally obtained data. UV-vis absorption spectra of L1, L2, L3, and L4 chromophores measured in dichloromethane (~C = 2.6 × 10^−5^ M) at room temperature.

The UV-vis electron absorption spectra were computed for LX molecules placed in dichloromethane using DFT/B3LYP and DFT/LC-BLYP methods and the obtained data were compared with experimental results (see Figure 16). One may conclude that the UV-vis spectra calculated with the DFT/LC-BLYP method better suit the experiments than the results obtained by DFT/B3LYP calculations. This is especially seen for the L3 and L4 molecules possessing higher dipole moments than L1 and L2 [34]. It is caused by the nature of the method and the important charge redistribution in molecules possessing 2,4-dinitrophenyl groups. In consequence, the NLO properties of the LX chromophores will be calculated using DFT/LC-BLYP formalism.

### 2.4. NLO Properties of the LX/PMMA Systems 

The quantum chemical calculations were performed to explain the phenomenon of the NLO peculiarities occurring in the LX/PMMA composites. The polarizabilities, as well as the first- and second-order hyperpolarizabilities, were computed by implementing the DFT/LC-BLYP method into the Dalton program [35]; the obtained data are presented in Table 3, Table 4, Table 5 and Table 6. Frequency-dependent polarizabilities and hyperpolarizabilities were computed with the random phase approximation (RPA) [36]. The NLO parameters were calculated for the L1, L2, L3, and L4 molecules in vacuum, and these data are presented in the column indicated by F_x_, F_y_, F_z_ = 0, 0, 0. When the column is described by the F_x_, F_y_, F_z_ ≠ 0, 0, 0 it means that the optical parameters were computed by applying a discrete local electric field. The optical parameters were calculated for the rigid molecules at their most stable geometry. In the work of Champagne et al. [37], it was reported that structural fluctuations have an important role in NLO response. In our work, this computational problem was reduced by calculating the NLO parameters of the two chromophore conformations. In this case, the representative sets of molecular geometries were used.

In Table 3, the optical parameters of the L1 molecule in **a** and **b** states were calculated by using the DFT/LC-BLYP method. The local electric field acting on the chromophores L1 located in PMMA is equal to 0.73 GV/m, with the highest intensity along the X-axis. The PMMA matrix does not affect the static and frequency-dependent polarizability of the L1 chromophores; however, it increases insignificantly the second- and third-order hyperpolarizabilities. The second-order hyperpolarizability (β_vec_) of the L1^b^ molecule is higher than that of the L1^a^ molecule, while the situation is the opposite with the hyperpolarizability γ_vec_. The L1^b^ has a lower γ_vec_ value than the L1^a^. 

The local electric field acting on L2 molecules embedded into the PMMA matrix is equal to 1.03 GV/m (see Table 4). It is the highest value of the local electric field calculated for the investigated TTF derivatives in PMMA. It is not the reason for the close location of the L2 molecules to the PMMA because the distance between L3 and L4 and the polymer is smaller. The high value of the local electric field is caused by the polarization of composite and dipolar interactions between guest and host structures. The PMMA matrix increases the electric dipole moment of the L2 chromophores, decreasing its HOMO–LUMO energy difference. However, the PMMA polymer does not have a significant impact on the polarizability of the L2 chromophores and almost does not affect their third-order NLO parameters. Notwithstanding, the PMMA drastically increases the second-order hyperpolarizability of the L2^b^ chromophores and decreases the β_vec_ of the L2^a^ molecules. The L2^b^ possesses the highest β_vec_ value of all of the investigated samples. Additionally, these chromophores are not sensitive to the back relaxation process. All these facts have an impact on the highest second-order susceptibility of the L2 chromophores embedded into the PMMA matrix. Also, the γ_(z;z,z,z)_ of the L2^b^ has an important value compared with other molecules affected by the PMMA matrix even if it is not enhanced by the polymer.

The local electric field acting on the L3 molecules embedded into the PMMA matrix is equal to 0.21 GV/m (see Table 5). It is the lowest value obtained for all investigated composites. One can see that the PMMA matrix is almost neutral for the L3 molecule in both conformers calculating linear optical properties. Polarizability values calculated in vacuum and with local electric field (F_x_, F_y_, F_z_ ≠ 0, 0, 0) are almost the same. However, the PMMA matrix has a destructive effect on the NLO parameters of L3 molecules. The L3/PMMA composite will not be suitable for NLO applications; however, its γ_(z;z,z,z)_ value is relatively high compared with other molecules.

The local electric field calculated for the L4 molecules embedded into the PMMA matrix is equal to 0.57 GV/m (see Table 6). The PMMA environment increases the dipole moment of both conformers by more than 1 D, decreasing the HOMO–LUMO difference of conformer **a** and increasing this value for conformer **b**. The PMMA matrix increases the second-order susceptibility of the L4 molecule in both conformers, while almost not changing their third-order hyperpolarizability. One should note that the L4^b^ has a very low β_vec_ compared to that of L4^a^. This molecule will not give a significant signal of the SHG by taking into consideration the β_vec_ quantity and tendency to the back relaxation of the L4 chromophores in PMMA. Environmental effects have a distinguishable impact on the first hyperpolarizability, as was shown in the work of Fronseca et al. [19]. In conclusion, one can say that the intermolecular interaction occurring between PMMA and chromophores enhanced the SHG signal for L1, L2, and L4 molecules. The PMMA matrix has a destructive influence on the NLO parameters of L3 chromophores. It was confirmed in our previous work [38].

The NLO properties of the host–guest systems based on the studied TFF derivatives and PMMA polymer matrix were also investigated experimentally. Measurements of the SHG and THG signals and sample preparation in detail are described in our previous work [18]. The concentration of chromophores in PMMA was 2 wt%. We acknowledge the importance of chromophore content in determining the effectiveness of NLO materials. The materials studied experimentally have been optimized to achieve high chromophore loading while maintaining stability and homogeneity. This optimization ensures that the NLO properties are maximized without compromising the structural integrity of the composites. The careful balance of high chromophore content with an appropriate polymer matrix is crucial for enhancing the NLO parameter values. The investigated materials not only match but often surpass the NLO performance of traditional reference materials. 

The obtained values of the χ^(2)^ and χ^(3)^ susceptibilities are presented in Table 7. Analyzing these data, one can see that the highest SHG signal was measured for the L2/PMMA composite but the lowest signal was obtained for L3/PMMA. It confirms the correctness of the performed calculations presented above. The L2 chromophores embedded in the PMMA matrix act by high β_vec_ and, additionally, the poled composites are very stable and do not undergo rapid back relaxation. This results in the high SHG signal coming from L2/PMMA.

The low value of the χ^(2)^ measured for L3/PMMA is also in agreement with the performed calculations. The PMMA matrix has a destructive effect on the β_vec_ calculated for L3, giving a low second-order NLO signal. Additionally, the poled L3/PMMA composite is unstable concerning the chromophore dipole alignment that does not have a positive effect on SHG generation. The SHG signal is similar for the L1/PMMA and L4/PMMA, which means that the 2,4-dimethylphenyl or 2,4-dinitrophenyl groups accompanied by the TTF group attached to the pyridyl ring in orto-position do not affect the SHG signal.

The obtained THG signal is not significant for all composites, giving the values χ^(3)^ from 1.60 × 10^−21^ m^2^V^−2^ for L3/PMMA up to 1.80 × 10^−21^ m^2^V^−2^ for L2/PMMA. In our previous work, we reported THG data obtained for LX-based thin films performed by the pulsed laser deposition (PLD) technique [18]. It was seen that the L3 film shows a much higher THG response compared to L1, L2, and L4 films. It confirms the statement that a higher third-order NLO signal obtained for PLD-prepared thin films of L3 comes from inter-chromophore interaction and it is the effect of the intrinsic properties of the L3 molecules. The γ_vec_ of L3^a^ is high and, additionally, when enhanced by inter-chromophore interaction, it gives a high THG signal. This interaction is not seen in the L3/PMMA composite. Regarding the χ^(2)^ values for the studied composites, it is important to note that they are comparable or significantly higher than those of well-known reference materials such as Y-cut quartz (χ^(2)^ = 1.0 × 10^−12^ mV^−1^) [39]. The measured χ^(3)^ for investigated materials are in the range of and are much higher than the respective value of the reference silica material (χ^(3)^ = 0.20 × 10^−21^ m^2^V^−2^) [40]. This comparison underscores the potential of our materials in practical applications (Table 7).

The guest–host composites based on PMMA and LX molecules give a lower signal of THG than the PLD materials. It is probably due to the aggregation effect that cancels the intensity of the dipole moment or the interaction with the polymeric matrix is destructive in this case. This observed effect is especially seen for the L4 and L3 compounds with high dipole moments. One can conclude that the nature of the intermolecular interaction and their influence on the NLO properties of the LX/PMMA composites come from intermolecular electrostatic and dipole interactions as well. The polymer matrix can significantly influence the NLO properties of the composite. Depending on its interaction with the guest molecules, the polymer matrix can either enhance or diminish the overall NLO response. This interaction can affect factors such as the alignment, distribution, and orientation of the guest molecules within the matrix, which are crucial for optimizing the desired NLO effects. Therefore, careful selection and optimization of both the guest molecules and the host matrix are essential to achieve the best performance in practical applications. One can see that the L2 and PMMA components are well suited to construct NLO devices. Unfortunately, the L3 and PMMA components cannot be used together. The performed computer simulations based on a hierarchical model explain the phenomena observed experimentally.

## 3. Calculation Methods

### 3.1. Molecular Dynamics Simulations 

The molecular dynamics (MD) method is used in the presented work to determine the structure of the bulk (volumetric) and thin film composite materials based on LX molecules embedded into the host PMMA polymer matrix. Therefore, four different composite systems were created, namely L1/PMMA, L2/PMMA, L3/PMMA, and L4/PMMA. Three different starting structures were built for each of the guest–host systems using HyperChem software [41]. The unit cell of each investigated LX/PMMA composite consists of two chromophore molecules and one chain of the isotactic 90-mer PMMA with a molecular weight of 9012.58 amu. Every investigated unit cell is in cubic with an edge length of 24.8 Å and a density equal to 1.00 g/cm^3^ for each system. The required density corresponds to the liquid state of the investigated complexes. 

First of all, each initial structure was relaxed to reach the minimum of total energy by two methods following one another. First, the steepest descent algorithm employing a convergence criterion of 200 kcal mol^−1^Å^−1^ was used, and then the procedure was continued by the conjugate gradient method with a convergence criterion equal to 50 kcal mol^−1^Å^−1^. Then, the obtained structures were modeled using the MD method. The simulations were performed using the GROMACS software [42]. The non-bonded interactions were taken into account with the Lennard-Jones 12-6 potential and a Coulomb potential based on defined charges of atoms. Bonded interactions were computed due to a fixed list of atoms. Bond stretching and bond bending were presented by harmonic potentials, whereas dihedral angle distortions were modeled by a simple cosine function. The all-atom consistent valence force field (CVFF) [43] was selected for the MD modeling. The CVFF force field was used to simulate the structure and dynamic behavior of the host–guest systems with organic π-conjugated dyes as active molecules [29,44]. All MD simulations were performed with the following parameters: the simulation time step was 1 fs, and the short-range neighbor list was created employing the grid search method with a cut-off distance equal to 1.10 nm. The Lennard-Jones and Coulomb interactions were computed within the neighbor list by employing periodic boundary conditions. The long-range interactions were calculated using the three-dimensional particle-mesh Ewald method (PME) [45,46]. The energies, coordinates, and velocities of the atoms were recorded every one picosecond. The major part of the simulations was performed in the NVT canonical ensemble (the number of atoms, volume, and temperature were constant) employing the Nose–Hoover thermostat [47,48]. A simulated cooling process of the investigated composites was modeled in an NPT ensemble (the number of atoms, pressure, and temperature were constant) using the Rahman–Parrinello barostat [49] to control the pressure during simulations.

The LX/PMMA composites with optimized geometries were relaxed during 5.0 ns under NVT conditions at a temperature of 500 K. All relaxed structures reached the thermodynamic equilibrium after 1.5 ns of simulations. Then, the poling external electric field was applied in the Z-direction of the laboratory coordinate system and the composites were modeled again during 2.0 ns in the NVT ensemble. The strength of the applied external electric field used for the dipolar alignment of the chromophores was equal to 1, 3, 5, 10, and 15 kV/µm for all complexes modeled in bulk form. The method for selecting the magnitude of the external electric field is described in our previous work [29]. It should be noted that the experimentally commonly used electric field in the corona poling process is equal to 10 kV/cm. However, we want to achieve the equilibrated state of the modeled materials within the time scale of the performed simulations, and the intensity of the used external electric field should accordingly be higher. 

The composites poled at 500 K as the results of the final configurations of the low-density MD runs were used in the simulation of the cooling process from 500 K up to 300 K in the presence of the external poling electric field, employing the simulated annealing (SA) process available in the GROMACS program. The simulations were performed in the isobaric–isothermal ensemble with a protocol implemented in GROMACS, where the reference temperature was varied linearly. The cooling rate was equal to 1.3 × 10^11^ K/s. Then, the high-density ordered systems were simulated for 1.5 ns in NVT condition at 300 K, keeping the external electric field working. This simulation was performed to obtain thermodynamically equilibrated high-density systems at 300 K. Then, the stability and back relaxation of the chromophores in the PMMA polymer matrix were investigated. In this case, the composites were modeled during 1.5 ns in the NVT ensemble at 300 K without acting an external electric field.

Additionally, three equivalent molecular complexes were created for each LX/PMMA composite to model their thin film forms. These structures were composed of two chromophores, respectively, L1, L2, L3, or L4, and one isotactic 90-mer PMMA polymer chain in each unit cell as it was in the case of bulk form. The simulated box of the investigated thin film was equal to 26.25 Å × 26.25 Å × 78.75 Å. The employed unit cell edges provided the possibility to model infinite extensions along the X- and Y-axes and finite structures in the Z-direction. The geometry of each modeled atomic system was optimized by energy minimization using the steepest descent algorithm with a convergence criterion of 100 kcal mol^−1^Å^−1^ and then by the conjugate gradient method, employing a convergence criterion of 20 kcal mol^−1^Å^−1^. The MD simulations were carried out at 500 K and 300 K with a 1 fs size step. All the remaining parameters were the same as employed for the MD of bulk composites. The long-range non-bonded interactions were calculated using the 2D PME method. The values of the external poling electric field were 0.5, 1, 3, and 5 kV/μm. The systems were solidified with a cooling rate of 1.3 × 10^11^ K/s. The final density for each film was 1.173 ± 0.005 g/cm^3^, corresponding to the experimental value of the glassy state of PMMA. The methodology of the performed MD simulations of thin films was exactly like that for the bulk materials, as presented above.

### 3.2. Parameters of Quantum Chemical Calculations 

The electron and optical properties of the LX chromophores were investigated computationally by applying the DFT method. First of all, their geometries were optimized upon the energy minimization procedure by applying the DFT formalism within the GAMESS program package [50]. The minimum of the potential energy surface was calculated in C1 symmetry at the restricted Hartree–Fock (RHF) level with B3LYP functional and the 6-311G** basis set, according to the work of Srinivasan et al. [51]. The quadratic approximation (QA) optimization algorithm based on augmented Hessian techniques was used to achieve the geometry of the studied molecules with the minimum of their total energy. The gradient convergence tolerance was equal to 10^−6^ Hartree/Bohr. After that, the optimized structures were used to calculate their electron and optical properties. The calculations were performed in GAMESS using B3LYP and LC-BLYP functionals with the 6-311++G** basis set for the isolated molecules placed in a vacuum or dichloromethane (CH_2_Cl_2_). The RHF SCF energy convergence criterion was chosen to be 10^−12^ Hartree. The separation parameter μ in the case of LC-BLYP functional was equal to 0.33. The Polarizable Continuum Model (PCM) [52] was used to investigate the solvent effect. The position and oscillator strength values for the electron-excited states of the LX molecules were calculated using the iterative Davidson method [53] with an accuracy of 10^−12^ Hartree. The calculations were performed using the time-dependent DFT (TDDFT) formalism in the vertical approach according to the works of Jacquemin et al. [54,55]. We are aware that these are approximate calculations and the results are not as precise as those obtained in the adiabatic approximation [56], but they can help to interpret the polymeric matric effect on the optical properties of LX molecules. For each molecule, the 15 first excited states were calculated. The polarizabilities and hyperpolarizabilities of the LX molecules were calculated using the DFT/LC-BLYP method with the 6-311++G** basis set implemented in the Dalton program (Dalton2020.0.beta (2020), http://daltonprogram.org, accessed on 6 March 2022) [35]. Frequency-dependent parameters were computed with the random phase approximation (RPA) [36]. 

In the framework of local field theory [57], linear and nonlinear macroscopic susceptibilities are related to molecular properties by local field factors, which describe the effect of the electric field on a molecular site induced by the neighboring atoms. In discrete local field theory, the local fields are computed by considering the molecular environment rigorously, without resorting to a continuum or mean field approximations. The simplest scheme used corresponds to the point dipole approximation, where the electrical response of each chromophore molecule and each monomer entity of the PMMA chain to the local fields induced by the externally applied optical fields are expressed by induced point dipoles situated in the respective chromophore center of mass (COM). Because of the intramolecular flexibilities of each molecule in the MD simulation, the COM of each molecule was determined anew at each considered snapshot of the trajectories. 

Local field theory takes into account the effect due to the local field F acting on the molecule. In the case where partial charges q_Ns_ are assigned to atomic sites s, as in the MD simulations whose trajectories will be used here, the components of this field can be calculated by the following formula:

(2) $$F_{Nsi}={\left(\epsilon_{0}\upsilon \right)}^{-1}\sum_{s\prime }L_{si,s\prime }q_{s\prime }$$

where the $L_{si,s\prime }$ are components of dipole-charge lattice sums, giving the Coulomb field at site s due to all charges at site s′ [58,59]. Equation (2) does not include the induced fields, which generally leads to an underestimation of the field effect. However, in our case, the polarizability of the polymer is rather small and the more strongly polarizable chromophore molecules are too far apart to influence each other considerably.

To calculate the local field F(x,y,z) defined by Formula (2), the poled LX/PMMA composite structures modeled by MD simulations were considered. The local electric field was calculated over trajectories of each of the three MD-modeled L1/PMMA, L2/PMMA, L3/PMMA, and L4/PMMA structures and the results were averaged. The sites s’ denote all atoms surrounding the molecule of interest (sites s) in the modeled unit cell and the next coordination spheres of unit cells of the composite up to the moment when the calculated F is saturated. The chosen difference between F_n_-F_n−1_ is taken to be not higher than 10^−4^ GV/m. The obtained local field F(x,y,z) was included into the Hamiltonian to calculate polarizabilities and hyperpolarizabilities affected by the polymeric matrix. To have the possibility to compare data obtained for different systems, they were rotated to have the electric dipole moment of the discussed LX molecule along the direction of Z-axis in laboratory coordinates.

The optical properties of the chromophores were computed only for the optimized geometry, and it was assumed that the optical parameters were the same for each geometric structure adopted during the simulations.

## 4. Conclusions

In the present work, the guest–host composites based on the PMMA polymer and LX chromophores in the bulk and thin film form were investigated. The structural properties of the modeled systems obtained by MD simulations were discussed. The influence of the external poling electric field on the chromophores alignment was studied. Also, their optical parameters were measured experimentally and determined from quantum chemical calculations. 

Analyzing the MD simulation results, one can conclude that the L3 and L4 molecules enter more deeply into the bulk PMMA polymer structure than the L1 and L2 molecules. This behavior is significantly visible for the system in the glassy state (at T = 300 K). It can be inferred that the chromophores possessing the 2,4-dinitrophenyl groups tend to be located more between polymer mers than the chromophores with 2,4-dimethylphenyl groups. Additionally, one can add that the meta-position of the TTF group on the pyridine tends to be closer to the location of the chromophores to a polymer chain. 

In the case of polymer thin film, all chromophores are located at a distance from the PMMA of about 0.4 nm. The chromophores do not enter between mers. It means that the surface of the polymer is less dense than it is observed for bulk guest–host composite systems. It has a great influence on the chromophore alignment under the influence of the external electric field. It is much easier to align the chromophores in the thin film PMMA than in the bulk form. 

The L2 chromophores in the bulk guest–host system are the easiest to align compared to the remaining chromophores. The molecules L3 and L4 are significantly more difficult to align than the L1 and L2 chromophores. This is what happens even though the molecules L3 and L4 possess much bigger electric dipole moments than the L1 and L2 chromophores and their interaction with the external electric field is much higher. The difficult alignment of the molecules L3 and L4 should be caused by their location in the polymer cages where rotation is difficult. However, the structure of composites L3/PMMA and L4/PMMA are more stable concerning the back relaxation of the chromophores.

The electron and optical properties of the LX chromophores were investigated computationally by applying the DFT method. These chromophores embedded into the PMMA matrix were also studied experimentally on account of their optical properties. Optimizing the geometry of the LX molecules, two conformers of each one were found. Additionally, it was found that the probability of finding chromophores in both configurations is almost the same. The results obtained for the L2/PMMA guest–host system are encouraging for their different NLO applications like optical communications and optical switching.

## Figures and Tables

**Table 1:** Structures and frontier molecular orbitals calculated for L1, L2, L3, and L4 molecules by DFT/B3LYP/6-311++G** methodology.

	Conformer a		Conformer b	
	HOMO	LUMO	HOMO	LUMO
**L1**	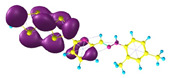	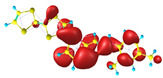	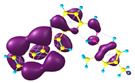	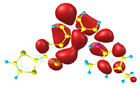
**L2**	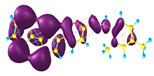	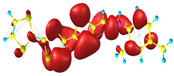	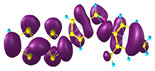	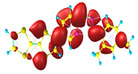
**L3**	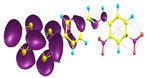	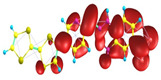	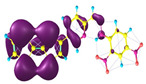	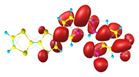
**L4**	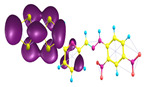	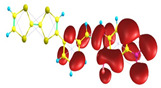	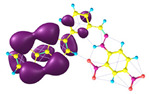	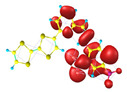

**Table 2:** Electron and optical properties of the L1, L2, L3, and L4 molecules in conformation a and b calculated in vacuum and dichloromethane by DFT/B3LYP and DFT/LC-BLYP methods.

Molecule	ΔE_HOMO-LUMO_ [eV]		Dipole Moment [D]		λ_max_ [nm]		λ_max_ [nm]	
	In Vacuum						In Dichloromethane	
	B3LYP	LC-BLYP	B3LYP	LC-BLYP	B3LYP	LC-BLYP	B3LYP	LC-BLYP
**L1** ^a^	3.23	6.90	1.24	1.00	500.8	409.6	500.1	408.7
**L1** ^b^	3.13	7.31	2.55	2.95	509.0	415.4	500.1	409.1
**L2** ^a^	2.94	6.55	1.56	1.12	520.1	415.2	518.1	414.7
**L2** ^b^	2.85	6.89	2.72	3.30	542.2	423.8	534.2	416.9
**L3** ^a^	1.99	6.16	6.66	6.03	717.5	416.1	718.8	409.3
**L3** ^b^	2.13	6.27	10.56	9.96	680.5	422.9	737.1	415.6
**L4** ^a^	1.92	6.15	6.60	5.92	744.2	416.3	719.6	409.3
**L4** ^b^	1.68	5.90	8.39	8.10	971.7	416.0	891.3	423.9

**Table 3:** Optical properties of the L1 molecules calculated in vacuum (F_x_, F_y_, F_z_ = 0, 0, 0) and PMMA polymer environment (F_x_, F_y_, F_z_ ≠ 0, 0, 0) in volumetric form by using the DFT/LC-BLYP/6-31++G** method.

Molecule	L1^a^		L1^b^	
**F_x_**, **F_y_**, **F_z_** **[GV/m]**	**0**, **0**, **0**	0.63, −0.26, −0.27	0, 0, 0	0.63, −0.26, −0.27
***λ* = ** **∞ nm**				
**α**_vec_	348.22	348.63	340.75	341.15
**β**_(z;z,z)_	−234.12	−281.44	−993.81	−1192.27
**β**_(z)_	513.90	513.06	−1023.06	−1237.10
**β**_vec_	−1065.28	−1299.30	−1084.00	−1376.59
**γ**_(z;z,z,z)_	316,544.26	323,376.63	308,817.70	323,428.03
**γ**_vec_	152,423.09	156,069.64	125,806.73	129,839.36
***λ* = 1064 nm**				
**α**_vec_	354.97	355.43	347.15	347.62
**β**_(z;z,z)_	−234.54	−286.65	−1803.34	−2134.06
**β**_(z)_	820.20	842.96	−1876.65	−2211.48
**β**_vec_	−1529.15	−1842.83	−1964.15	2387.40
**γ**_(z;z,z,z)_	780,156.42	808,763.27	1,037,673.87	1,153,715.81

**Table 4:** Optical properties of the L2 molecules calculated in vacuum (F_x_, F_y_, F_z_ = 0, 0, 0) and PMMA polymer environment (F_x_, F_y_, F_z_ ≠ 0, 0, 0) in volumetric form by using the DFT/LC-BLYP/6-31++G** method.

Molecule	L2^a^		L2^b^	
**F_x_**, **F_y_**, **F_z_** **[GV/m]**	**0**, **0**, **0**	1.00, 0.08, −0.24	0, 0, 0	1.00, 0.08, −0.24
***λ* = ** **∞ nm**				
**α**_vec_	350.71	350.10	355.06	355.30
**β**_(z;z,z)_	1044.65	537.31	−2168.85	−2639.76
**β**_(z)_	1523.84	724.76	−2292.25	−2819.59
**β**_vec_	1135.96	648.52	−2265.53	−2780.98
**γ**_(z;z,z,z)_	343,711.47	330,617.13	767,234.87	791,318.81
**γ**_vec_	180,307.29	176,382.50	220,527.36	225,157.26
***λ* = 1064 nm**				
**α**_vec_	357.80	357.12	363.20	363.48
**β**_(z;z,z)_	1572.70	777.96	−4137.86	−4948.54
**β**_(z)_	2217.61	981.05	−4330.76	−5222.10
**β**_vec_	1788.29	906.73	4280.09	−5153.42
**γ**_(z;z,z,z)_	993,705.63	945,275.89	3,724,893.11	3,973,896.27

**Table 5:** Optical properties of the L3 molecules calculated in vacuum (F_x_, F_y_, F_z_ = 0, 0, 0) and PMMA polymer environment (F_x_, F_y_, F_z_ ≠ 0, 0, 0) in volumetric form by using the DFT/LC-BLYP/6-31++G** method.

Molecule	L3^a^		L3^b^	
**F_x_**, **F_y_**, **F_z_** **[GV/m]**	**0**, **0**, **0**	0.02, 0.13, −0.16	0, 0, 0	0.02, 0.13, −0.16
***λ* = ** **∞ nm**				
**α**_vec_	371.32	371.13	371.19	371.06
**β**_(z;z,z)_	1834.58	1533.67	1631.59	1537.15
**β**_(z)_	2686.05	2368.38	2458.76	2320.57
**β**_vec_	2809.73	2510.19	2533.30	2404.97
**γ**_(z;z,z,z)_	1,059,995.74	1,038,024.70	448,094.58	445,438.97
**γ**_vec_	272,530.64	267,830.58	179,103.70	242,816.49
***λ* = 1064 nm**				
**α**_vec_	380.51	380.28	380.42	380.33
**β**_(z;z,z)_	3403.30	2892.62	2336.48	2178.77
**β**_(z)_	4565.24	4030.50	3773.85	3544.41
**β**_vec_	4705.35	4189.16	3862.52	3644.54
**γ**_(z;z,z,z)_	4,263,921.80	4,065,808.73	1,758,796.83	1,738,381.81

**Table 6:** Optical properties of the L4 molecules calculated in vacuum (F_x_, F_y_, F_z_ = 0, 0, 0) and PMMA polymer environment (F_x_, F_y_, F_z_ ≠ 0, 0, 0) in volumetric form by using the DFT/LC-BLYP/6-31++G** method.

Molecule	L4^a^		L4^b^	
**F_x_**, **F_y_**, **F_z_** **[GV/m]**	**0**, **0**, **0**	0.03, 0.37, 0.43	0, 0, 0	0.03,0.37, 0.43
***λ* = ** **∞ nm**				
**α**_vec_	362.74	363.61	351.66	351.78
**β**_(z;z,z)_	197.62	576.51	664.40	886.57
**β**_(z)_	1440.12	2015.46	705.52	978.04
**β**_vec_	1698.35	2324.69	446.08	894.45
**γ**_(z;z,z,z)_	355,253.54	366,340.05	288,943.31	296,072.59
**γ**_vec_	174,379.43	182,956.26	137,652.19	137,585.37
***λ* = 1064 nm**				
**α**_vec_	370.63	371.61	358.77	358.91
**β**_(z;z,z)_	528.86	1117.53	983.49	1321.85
**β**_(z)_	2258.99	3146.95	1138.44	1627.22
**β**_vec_	2601.77	3667.28	943.73	1460.30
**γ**_(z;z,z,z)_	1,018,411.52	1,093,392.47	814,810.82	848,786.26

**Table 7:** Susceptibilities χ^(2)^ and χ^(3)^ measured experimentally for L1/PMMA, L2/PMMA, L3/PMMA, and L4/PMMA thin films.

Sample	χ^(2)^ [10^−12^ mV^−1^]	χ^(3)^ [10^−21^ m^2^V^−2^]
**L1**	1.10	1.70
**L2**	2.10	1.80
**L3**	0.79	1.60
**L4**	1.20	1.70
**Y-cut quartz**	1.00	
**Silica glass**		0.20

## Data Availability

Computationally obtained data are available from L.M. on personal request. The samples and experimentally obtained data are available from A.E.-G. and A.M.-Z. on personal request.
